# Changes in antimicrobial resistance in Salmonella enterica serovar typhimurium.

**DOI:** 10.3201/eid0604.000429

**Published:** 2000

**Authors:** F J Angulo, P M Griffin


3. Glynn MK, Bopp C, Dewitt W, Dabney P, Mokhtar M, Angulo FJ. Emergence of multidrug-resistant *Salmonella enterica* serotype typhimurium DT104 infections in the United States. N Engl J Med 1998;338:1333-8.
4. Smith KE, Besser JM, Hedberg CW, Leano FT, Bender JB, Wicklund JH, et al. Quinolone-resistant *Campylobacter jejuni* infections in Minnesota, 1992-1998. Investigation team. N Engl J Med 1999;340:1525-32.
5. European Commission (1999). Opinion of the Scientific Steering Committee on Antimicrobial Resistance (SSCOAR). European Commission Directorate General XXIV, Consumer Policy and Consumer Health protection, 28 May 1999.
6. Report of the Joint Expert Advisory Committee on Antibiotic Resistance (JETACAR). The use of antibiotics in food-producing animals: antibiotic-resistant bacteria in animals and humans. Australian Commonwealth Department of Health and Aged Care and the Commonwealth Department of Agriculture, Fisheries and Forestry; 1999 Sep; Canberra.
7. Turnidge J, Nimmo G, Francis G. Evolution of resistance in *Staphylococcus aureus* in Australian teaching hospitals. Australian Group on Antimicrobial Resistance (AGAR). Med J Aust 1996;164:68-71.
8. Collignon P, Bell J. Drug-resistant *Streptococcus pneumoniae*: the beginning of the end for many antibiotics? Australian Group on Antimicrobial Resistance (AGAR). Med J Aust 1996;164:64-7.

### Changes in Antimicrobial Resistance in *Salmonella enterica* Serovar Typhimurium

**To the Editor:** The conclusion by Davis and colleagues (1) that use of antimicrobial agents in agriculture is unlikely to have contributed to the emergence of multidrug-resistant *Salmonella* serotype Typhimurium DT104 (MR-DT104) is contrary to available evidence. Use of antimicrobial agents in aquaculture in Asia may have contributed to the emergence of DT104. The resistant determinants of MR-DT104 reside on the chromosome, apparently within a transferrable element (2-4). Chloramphenicol resistance in MR-DT104 is due to *floR*, a florfenicol resistance gene (5); florfenicol is a veterinary antimicrobial agent that, although not approved in the United States until 1996, has been used in aquaculture in Asia since the early 1980s. *FloR* was first identified in *Photobacterium damsela,* a bacterium found in fish (5). Furthermore, tetracycline resistance in MR-DT104 is due to a class G resistance gene first identified in *Vibrio anguillarum*, a pathogen of fish (4,6). The molecular sequence where the class G and *floR* determinants reside on the DT104 chromosome is closely related (94% identity) to a plasmid in *Pasteurella piscicida*, another pathogen of fish (7). These data suggest that the resistance determinants of MR-DT104 may have emerged among bacteria in aquaculture and been horizontally transferred to *S.* Typhimurium DT104.

Spread of MR-DT104 between regions during international travel, as Davis and colleagues suggest, is unlikely because in industrialized countries *Salmonella* is seldom transmitted from person to person (8). Once MR-DT104 emerged, it spread rapidly to many regions through unknown means. The rapid emergence of MR-DT104 suggests a means of spread more efficient than person-to-person transmission. Possibilities include movement of infected breeding or "multiplier" stock or shipment of contaminated feed ingredients; such movements may not be as limited as Davis et al. suggest. For example, the international spread of *Salmonella* serotype Agona was traced to the global distribution of contaminated fish meal from Peru (9).

Once MR-DT104 is introduced into food animals in a region, use of antimicrobial agents in animals would contribute to further dissemination of MR-DT104 (8). If MR-DT104 is present on a farm, the use on the farm of any antimicrobial agent to which MR-DT104 is resistant would contribute to its persistence. An example of such use in cattle in the United States is the tetracycline-containing milk "replacement" commonly fed to dairy calves. This product could kill susceptible gastrointestinal flora while allowing tetracycline-resistant flora such as MR-DT104 to survive and proliferate. Once MR-DT104 proliferates on a farm, dissemination to other farms in the region is facilitated, particularly if the other farms are using an antimicrobial agent to which MR-DT104 is resistant.

Increasing antimicrobial resistance in *Salmonella* contributes to its spread and threatens the use of clinically important antimicrobial agents. To slow the emergence and dissemination of resistant *Salmonella,* measures should be implemented to ensure that antimicrobial agents are used prudently in food-producing animals (10).

**Frederick J. Angulo and Patricia M. Griffin**
Centers for Disease Control and Prevention,
Atlanta, Georgia, USA

### References

1. Davis MA, Hancock DD, Besser TE, Rice DH, Gay JM, Gay C, et. al. Changes in antimicrobial resistance among *Salmonella enterica* serovar Typhimurium isolates from humans and cattle in the Northwestern United States, 1982-1997. Emerg Infect Dis 1999;5:802-6.
2. Sandvang D, Aarestrup FM, Jensen LB. Characterisation of integrons and antibiotic resistance genes in Danish multiresistant *Salmonella enterica* Typhimurium DT104. FEMS Microbiol Let 1998;160:37-41.
3. Ridley A, Threlfall EJ. Molecular epidemiology of antibiotic resistance genes in multiresistant epidemic *Salmonella typhimurium* DT104. Microb Drug Resist 1998;4:113-8.
4. Briggs CE, Fratamico PM. Molecular characterization of an antibiotic resistance gene cluster of *Salmonella typhimurium* DT104. Antimicrob Agents Chemother 1999;43:846-9.
5. Bolton LF, Kelly LC, Lee MD, Fedorka-Cray PI, Maurer H. Detection of multidrug-resistant *Salmonella enterica* serotype *typhimurium* DT104 based on a gene which confers cross-resistance to florfenicol and chloramphenicol. J Clin Microbiol 1999;37:1348-51.
6. Zhao J, Aoki T. Nucleotide sequence analysis of the class G tetracycline resistance determinant from *Vibrio anguillarum*. Microbiol Immunol 1992;36:1051-60.
7. Kim EH, Aoki T. Drug resistance and broad geographical distribution of identical R plasmids of *Pasteurella piscicida* isolated from cultured yellowtail in Japan. Microbiol Immunol 1993;37:103-9.
8. Cohen ML, Tauxe RV. Drug-resistant *Salmonella* in the United States: an epidemiologic perspective. Science 1986;234:964-9.
9. Clark GM, Kaufmann AF, Gangrosa EJ. Epidemiology of an international outbreak of *Salmonella agona*. Lancet 1973;490-3.
10. Centers for Veterinary Medicine, U.S. Food & Drug Administration. Proposed framework for evaluating and assuring the human safety of the microbial effects of antimicrobial new animal drugs intended for use in food-producing animals. Washington: FDA; 1999 Jan 6. Available from: URL:http//www.fda.gov/cvm/fda/infores/vmac/antim18.htm

### Reply to Drs. Angulo and Collignon

**To the Editor:** Drs. Angulo and Collignon point out that exposure to one antimicrobial drug (e.g., tetracycline) can confer a selective advantage to a multiresistant organism (e.g., R-type ACSSuT) over nonresistant organisms. However, tetracycline use would not be expected to favor one tetracycline-resistant organism (MR-DT104) over other tetracycline-resistant organisms, and most bovine Typhimuriums before the MR-DT104 epidemic were tetracycline-resistant R-type ASSuT. Since neither florfenicol nor chloramphenicol was then available for use in livestock in the United States, the evidence suggests that the emergence of MR-DT104 in cattle populations was not driven by antibiotic selection pressure. The references Drs. Angulo and Collignon cited to establish the importance of antimicrobial use in livestock for the dissemination of multiresistant clones either do not address the issue of dissemination (1-2) or present evidence to the contrary: the dissemination of fluoroquinolone-resistant MR-DT104 despite the lack of fluoroquinolone use in the herds in question (3).

Available data support Dr. Collignon's example of *Campylobacter* as an agent for which fluoroquinolone use in livestock resulted in increasing prevalence of fluoroquinolone resistance (4). However, the epidemiology of resistance in polyclonal commensals such as *Campylobacter* is very unlike that of epidemic, clonal *S.* Typhimuriums. The epidemic, clonal dissemination of *S.* Typhimurium more closely resembles that of methicillin-resistant *Staphylococcus aureus* (MRSA). Epidemic MRSA clones differ genetically from nonepidemic ones, and dissemination of epidemic clones does not necessarily require antimicrobial selection pressure (5). Because antimicrobial usage practices that contribute to the control of MRSA have not been scientifically defined, infection control practices must play the central role in successful MRSA control programs (6-8).

Dr. Angulo's hypothesis that MR-DT104 emerged genetically in Asian fish is plausible, but other credible hypotheses exist. *tet*(G), first described in *Vibrio angullarium*, also occurs in *Pseudomonas aeruginosa* (9). Similarly, *flo*R is closely related to the *P. aeruginosa* chloramphenicol-resistance gene *cmlA* (10), and *pse*-1 encoded beta-lactamase is a common feature of hospital *P. aeruginosa* isolates (11). Thus, the hypothesis that MR-DT104 acquired resistance genes horizontally from nosocomial pseudomonads might also be worthy of consideration.

